# Modulation of physicochemical properties and antimicrobial activity of sodium alginate films through the use of chestnut extract and plasticizers

**DOI:** 10.1038/s41598-023-38794-3

**Published:** 2023-07-17

**Authors:** Weronika Janik, Michał Nowotarski, Kerstin Ledniowska, Divine Yufetar Shyntum, Katarzyna Krukiewicz, Roman Turczyn, Ewa Sabura, Simona Furgoł, Stanisław Kudła, Gabriela Dudek

**Affiliations:** 1 Łukasiewicz Research Network—Institute of Heavy Organic Synthesis “Blachownia”, 47-225 Kędzierzyn-Koźle, Poland; 2 Department of Physical Chemistry and Technology of Polymers, PhD School, Silesian University of Technology, 44-100 Gliwice, Poland; 3 Department of Physical Chemistry and Technology of Polymers, Faculty of Chemistry, Silesian University of Technology, 44-100 Gliwice, Poland; 4 Biotechnology Centre, Silesian University of Technology, 44-100 Gliwice, Poland; 5 Centre for Organic and Nanohybrid Electronics, Silesian University of Technology, 44-100 Gliwice, Poland

**Keywords:** Biomaterials, Green chemistry, Materials chemistry

## Abstract

Due to the growing demand for robust and environmentally friendly antimicrobial packaging materials, biopolymers have recently become extensively investigated. Although biodegradable biopolymers usually lack mechanical properties, which makes it inevitable to blend them with plasticizers. The purpose of this study was to investigate plasticization efficiency of bio-based plasticizers introduced into sodium alginate compositions containing chestnut extract and their effect on selected film properties, including primarily mechanical and antibacterial properties. The films were prepared by the casting method and sodium alginate was cross-linked with calcium chloride. Six different plasticizers, including three commercially available ones (glycerol, epoxidized soybean oil and palm oil) and three synthesized plasticizers that are mixtures of bio-based plasticizers, were used to compare their influence on the film properties. Interactions between the polymer matrix and the plasticizers were investigated using Fourier transform infrared spectroscopy. The morphological characteristics of the films were characterized by scanning electron microscopy. Thermal properties, tensile strength, elongation at break, hydrophilic, and barrier properties of the obtained films were also determined. To confirm the obtaining of active films through the use of chestnut extract and to study the effect of the proposed plasticizers on the antibacterial activity of the extract, the obtained films were tested against bacteria cultures. The final results showed that all of the obtained films exhibit a hydrophilic character and high barrier effect to oxygen, carbon dioxide and water vapor. In addition, sodium alginate films prepared with chestnut extract and the plasticizer proposed by us, showed better mechanical and antimicrobial properties than the films obtained with chestnut extract and the commercially available plasticizers.

## Introduction

Recently, there has been a growing interest in innovative and environmentally friendly antimicrobial packaging materials based on biodegradable polymers that extend the shelf life of food^1^. Among various packaging materials, petroleum-based plastics are dominant, especially for their low production cost and very good mechanical properties. However, their non-biodegradable nature has become a serious threat to the environment^2^. An alternative to non-biodegradable plastics are materials based on polysaccharides and among them, those based on starch, chitosan and sodium alginate have been the most popular^3^.

Sodium alginate, which is extracted from brown seaweed, is one of the most popular biodegradable polymers. The chemical structure of alginate consists of β-D-mannuronic acid (M) and *β*-L-guluronic acid (G)^4^. The monomers in the polymer chains are arranged alternately in GG and MM blocks, along with MG blocks^5^. Sodium alginate has high compatibility with most anionic substances, film-forming ability and is fully biodegradable^6^. However, pure sodium alginate films exhibit high water solubility, relatively poor mechanical properties and weak antimicrobial activity, making it necessary to modify them for use in packaging^7,8^. An important property of alginates is their ability to react with multivalent metal cations, specifically calcium ions. The ions form connections between MM and GG blocks of the polymer, yielding a three-dimensional network^5^. The cross-linked sodium alginate lose their hydrophilic character, what improves its water barrier properties and reduces water solubility^9,10^. Mechanical properties are also significantly improved by cross-linking^11^, as well as by the use of proper plasticizers, which improve flexibility of the films and expand their potential applications^12^.

In general, unmodified sodium alginate films are very brittle and fragile, what limits their application^13^.The use of plasticizers results in an increase in elongation at break and a decrease in tensile strength of the films^14^. In the case of biodegradable films, it is also important that the plasticizer has to be eco-friendly, biodegradable and non-toxic. The most commonly used plasticizers are polyols, of which glycerol^15–20^ and sorbitol^21–24^ are the most often incorporated into the alginate matrix. Jost et al.^21^ compared the efficiency of glycerol and sorbitol as plasticizers (20–50 wt%) for alginate films, finding that both positively affected mechanical properties, but led to differences in barrier properties. Incorporation of glycerol into the matrix increased permeability of oxygen and water vapor, while sorbitol had no effect on barrier properties. In another study, Jost and Stramm^22^ investigated the effects of glycerol, sorbitoland triethanolamine (20–50 wt%) as plasticizers of alginate and cornstarch films and they found that glycerol was more effective than sorbitol in mechanical properties improvement, while triethanolamine appeared to be as effective as glycerol, but it had a different effect on barrier properties. Triethanolamine reduced the water vapor and oxygen permeability of the film, while glycerol led to their increase, and sorbitol had no effect at all. Among polyols, mannitol (12.5–50 wt%)^25^, diethylene glycol (20 wt%)^26^, isopropanol (30 wt%)^27^ or polyethylene glycol (10–30 wt%)^26–28^ were also used for the plastification of sodium alginate films. In addition to glycerol and sorbitol, Olivas and Barbosa-Canovas^23^ proposed fructose and polyethylene glycol (PEG-8000) as plasticizers (40 wt%). Glycerol, sorbitol and fructose led to a significant increase in elongation at break of investigated films, when compared to PEG-8000. Water vapor permeability for films with fructose and with sorbitol showed the lowest values, while with PEG-8000—the highest. It was also found that PEG-8000 was incompatible with alginate, what was evidenced by phase separation. On the other hand, Paixao et al.^29^ used glycerol (Gly), tributyl citrate (TC) and their mixture as plasticizers for sodium alginate (70%TC + 30%Gly, 60%TC + 40%Gly, 50%TC + 50%Gly, 30%TC + 70%Gly and 10%TC + 90%Gly). The addition of tributyl citrate made the film less hygroscopic, as it was confirmed by solubility and water vapor permeability tests. Tributyl citrate and its mixture with glycerol increased tensile strength of the films, with higher values observed at higher concentrations of glycerol. Films plasticized with tributyl citrate and its mixture with glycerol were opaque, while films with glycerol were transparent. Aadil and Jha^28^ prepared films based on alginate and lignin using three different plasticizers: glycerol, polyethylene glycol and epichlorohydrin (10–15 wt%). The film with glycerol showed higher solubility and swelling values compared to films with the other plasticizers. In contrast, the film with polyethylene glycol had the highest tensile strength. In addition, films based on alginate and lignin in the presence of epichlorohydrin showed the highest thermal stability and better physico-mechanical and UV light barrier properties, and could be successfully used in packaging and coating applications. Unfortunately, epichlorohydrin can be used at low concentrations only, as it is toxic at higher ones.

Besides modifying mechanical properties, one of the other desirable modification of alginate is to obtain packaging materials with antimicrobial properties. Such materials can significantly reduce the number of undesirable microorganisms in food. Antibacterial activity of sodium alginate-based films can be provided by adding to the polymer matrix various bioactive nanoparticles, such as ZnO, Ag, CuO, or natural extracts^30–33^. A wide range of such extracts can be found in the literature, including extracts of white ginseng^34^, green tea^35^, peanut red skin^36^ or roselle hibiscus^37^. In addition, antibacterial properties of alginate films can also be provided by tansy essential oil^38^, lemongrass essential oil^30^, basil leaf ethanol extract^39^ or brown propolis extract^40^. Chestnut extracts used as antibacterial agents in different polysaccharide-based films have been indicated by recent studies^41–45^ as well. Since chestnut extract is a rich source of polyphenolic compounds, phenolic acids, and tannins^46–48^, its presence in the packaging material is expected to provide antibacterial activity over a wide range of bacterial strains, either by a direct killing of bacteria or by attenuation of bacterial pathogenicity^49^. To be active, chestnut extract should migrate from the internal space of the material to its surface, to be in contact with bacterial cells. Since the addition of the plasticizer leads to higher free volume between polymer chains, it should also allow the chestnut extract particles to migrate and fully reveal the antibacterial properties. In consequence, it should be possible to modulate antibacterial properties of sodium alginate films by using deliberately selected plasticizers. In addition to the antimicrobial properties that nanomaterials bring to food packaging, concerns about the safety of nanomaterials should not be ignored. A number of researchers^50–55^ have investigated the problem of safety of using the nanomaterials to provide so-called active, or antibacterial, films. Studies have focused on the risk of migration of nanoparticles from packaging materials into food and their impact on consumer health^56^. It has been found that due to the small size, nanomaterials can bioaccumulate in the body's organs and tissues^53,57^. Extended migration studies are therefore needed before marketing antibacterial packaging with nano-additives. Even if a substance is GRAS (generally regarded as safe), additional testing is required to assess the risks associated with its nano counterparts, since the physicochemical properties of nanostates differ significantly from those of macrostates^53^. The aim of the present study was to investigate plasticization efficiency of bio-based plasticizers introduced into sodium alginate compositions containing chestnut extract and their effect on selected film properties, suitable for food packaging applications. Effects of six plasticizers (three commercially available, i.e. glycerol, epoxidized soybean oil and palm oil, and three mixtures synthesized by us: (i) esters of propylene glycol with acetic acid, (ii) esters of propylene glycol with oleic acid and succinic acid, (iii) epoxidized esters based on propylene glycol, oleic acid and succinic acid, on the mechanical and physicochemical properties, including moisture content, swelling degree, total soluble matter content and gas permeability (oxygen, carbon dioxide and water vapor) were studied. Structural changes (infrared spectroscopy) and thermal properties (thermogravimetric analysis) of the materials as well as surface topography of the films (scanning electron microscopy) were also investigated. To confirm the antimicrobial activity of the films prepared with chestnut extract and different plasticizers, microbiological tests were conducted with the use of *Escherichia coli*, *Staphylococcus epidermidis*, and *Candida albicans*.

## Materials and methods

### Materials

Sodium alginate (Brookfield viscosity 350–550 mPa × s, c = 1%, w/w at 20 °C) was supplied by Across Organics (Geel, Belgium). Chestnut extract Farmatan (≥ 76% tannins) was provided by Tanin Sevnica (Sevnica, Slovenia). Calcium chloride (purity ≥ 96%) and acetic acid (99.5–99.9%) were purchased from Avantor Performance Materials (Gliwice, Poland). Glycerol was supplied by Merck (Darmstadt, Germany), while epoxidized soybean oil and epoxidized palm oil were from Inbra Indústrias Químicas LTDA (Sao Paulo, Brasil) and Malaysian Palm Oil Board (Kajang, Malaysia), respectively. Propylene glycol, cyclohexene and toluene (both pure p.a.), formic acid (85.0%), hydrogen peroxide (30.0%), disodium hydrogen phosphate dihydrate and sodium hydrogen carbonate (pure p.a.) were purchased from Chempur (Piekary Śląskie, Poland). Oleic acid (90.0%) was supplied by Alfa Aesar (Ward Hill, MA, USA), and succinic acid (≥ 99.5%) by POL-AURA (Zabrze, Poland). Methanesulfonic acid (> 99.0%) was provided by TCI (Zwijndrecht, Belgium).

### Preparation of the bio-based plasticizer mixtures

Plasticizer mixtures were prepared as described in our previous study^58,59^. In short, esterification reactions (propylene glycol with acetic acid, propylene glycol with oleic acid and succinic acid) and epoxidation reactions (epoxidation of mixed esters based on propylene glycol, oleic acid and succinic acid) were carried out in a glass reactor of 500 or 1000 cm^3^ volume, equipped with a mechanical stirrer, a temperature controller, Dean-Stark trap (for esterification reactions), reflux condenser and dropping funnel (for selected reactions). The schematic route of the synthesis is shown in Figs. S1, S2 and S3 (Supplementary).

### Preparation of the antibacterial films

**Figure 1:**
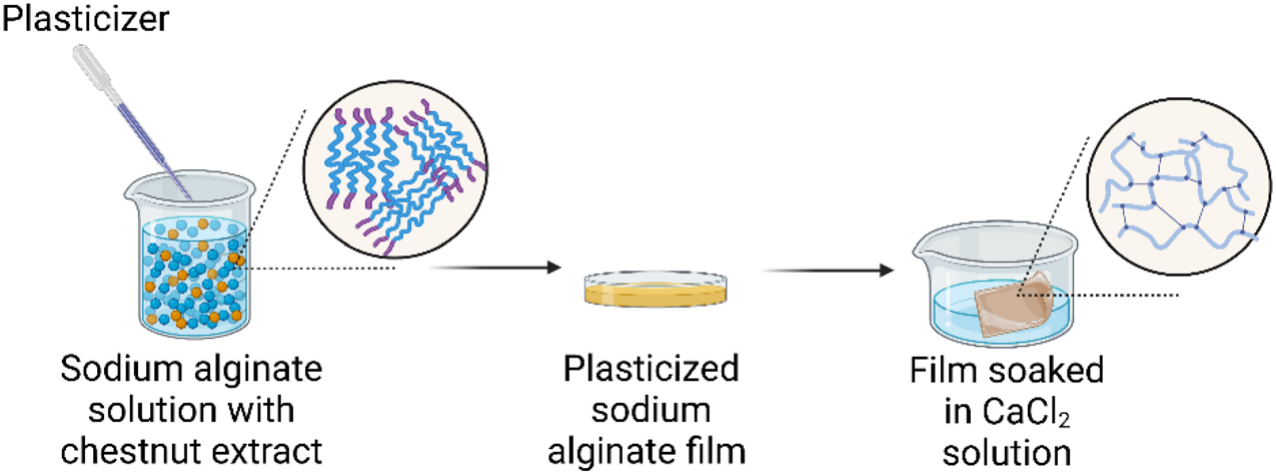
Preparation of plasticized and crosslinked sodium alginate films.

Sodium alginate films with chestnut extract and different plasticizers were prepared by casting method and then subjected to cross-linking reaction with calcium chloride (Fig. 1), according to the method described in our previous study^41^. In short, aqueous sodium alginate solution (1%, w/w) was prepared by dispersing sodium alginate with chestnut extract (0.75%, w/v) and a selected plasticizer (30%, w/w based on the mass of alginate)—Table 1.The films were obtained by casting the solution (46 g) onto Petri dishes (12 × 12 cm dish) thus obtaining a thickness of ca. 50 μm. After drying (24 h), the films were subjected to a cross-linking process. For this purpose, 40 ml of a 2.5% calcium chloride solution was poured onto the dried film and left on the film for 2 h under cover. After this time, the film was rinsed with distilled water and laid out on a paper towel, which was pressed to prevent the film from wrinkling during drying.

**Table 1:** Samples under investigations.

Symbol	CP1	CP2	CP3	MP1	MP2	MP3
Plasticizer	Commercial			Synthesized		
Description	Glycerol	Epoxidized soybean oil	Epoxidized palm oil	Mixed esters of propylene glycol and acetic acid	Mixed esters of propylene glycol, oleic acid and succinic acid	Epoxidized mixed esters of propylene glycol, oleic acid and succinic acid

### Mechanical properties

Mechanical properties i.e. tensile strength and elongation at break of sodium alginate films with chestnut extract and various plasticizers were examined using Instron 4466 testing machine. The analysis was performed on film samples 2 cm × 8 cm. The samples were stretched at a speed of 5 mm/min at room temperature. Final values were calculated as the average of ten measurements. During testing of the mechanical properties, the thickness of the film was measured using a digital micrometer (Mitutoyo Absolute Tester, Tokyo Sangyo Co. Ltd., Japan) with a resolution of 0.001 mm. The values presented were calculated as an average value of 10 measurements taken at different points for each sample.

### Hydrophilic properties

Hydrophilicity of sodium alginate films with chestnut extract and various plasticizers was analyzed using three-step gravimetric method (moisture content—MC, swelling degree—SD, and total soluble matter—TSM) and by determination of the contact angle values.. Film samples with surface area of 1 cm^2^ were weighed (M_1_), dried at 100 °C for 24 h and weighed again (M_2_).

1 $$MC\left( \% \right) = \frac{{\left( {M_{1} - M_{2} } \right)}}{{M_{1} }} \times 100$$

The samples were then placed in 30 mL of distilled water, left at room temperature for 24 h and weighed again (M_3_).

2 $$SD\left( \% \right) = \frac{{\left( {M_{3} - M_{2} } \right)}}{{M_{2} }} \times 100$$

In the final step, the samples were dried at 100 °C for 24 h and weighed (M_4_). Measurements were repeated five times and the average value was calculated. TSM values were calculated using the following formulae:

3 $$TSM\left( \% \right) = \frac{{\left( {M_{2} - M_{4} } \right)}}{{M_{2} }} \times 100$$

The water contact angle of the film surface was measured using an optical contact angle meter and a contour analysis system (OCA15 from DataPhysic). Droplets of distilled water (1 μL) were examined at ten points on each sample. All measurements were performed at ambient temperature (ca. 24 °C).

### Barrier properties

Oxygen and carbon dioxide permeability of the sodium alginate films with chestnut extract and various plasticizers were determined using an isobaric apparatus^58^. The samples with a circular surface area of 60 mm^2^ were degassed for 24 h and conditioned with the appropriate gas in the apparatus prior to testing for 2 h. Then, the diffusion chamber was sealed and compressed oxygen (class 5.0) or carbon dioxide (technical gas) was supplied at a controlled flow rate to keep the pressure constant. The permeation coefficient was determined as follows:

4 $$P = \frac{V \times l}{{S \times \Delta p}}$$

where V is the volumetric flow (mol s^−1^), l is the sample thickness (m), S is the sample area (m^2^) and Δp is the pressure difference on both sides of the sample (Pa).

Water vapor transmission rate (WVTR) and water vapor permeability (WVP) of sodium alginate films with chestnut extract and various plasticizers were determined according to the methodology proposed by Aguirre-Loredo et al.^60^ and Jiménez-Regalado et al.^61^. The samples, in the form of discs, were mounted on a glass container (internal diameter of 24.64 mm) with silica gel in its interior (~ 0% relative humidity, RH) and sealed with a liquid paraffin. After the paraffin solidified, the cup was weighed in order to calculate the initial weight. The covered glass container was then placed in a desiccator containing a supersaturated saline solution of BaCl_2_ (90% RH), generating a water-vapor differential pressure of 2854.23 Pa. The glass container was weighed seven times at 60 min intervals. The determinations were made in triplicate. The WVTR and WVP values were determined as follows:

5 $$WVTR = \frac{m}{tA}$$

6 $$WVP = WVTR \times {\text{L}}\Delta {\text{p}}$$

where ∆m/∆t is the moisture weight gain in time (g/s), A is the exposed surface area of the film (m^2^), L is the thickness of the film (mm), and ∆p is the difference in partial pressure (2854.23 Pa).

### Surface morphology

The morphology of the sodium alginate films with chestnut extract and various plasticizers was examined by a scanning electron microscopy (Phenom ProX) at 10 kV accelerating voltage, as well as an optical profilometer (Filmetrics Profilm 3D, KLA Co.). Surface roughness was obtained according to ISO 25178 via arithmetical mean height area roughness parameter (S_a_).

### Antimicrobial activity

Antimicrobial activity of sodium alginate films with chestnut extract and various plasticizers, as well as a control sample (sodium alginate film plasticized with glycerol, without chestnut extract) was determined against *Escherichia coli* ATCC25922, *Staphylococcus epidermidis* ATCC12228, and *Candida albicans* ATCC18804. The samples in the form of discs (10 mm in diameter) were placed in 12-well plates containing 200 µl of M9 minimal medium supplemented with glucose as the sole carbon source. Thereafter, 20 µl of the targeted bacterial culture, normalized to 10^4^ CFU/ml, was inoculated into each well and incubated overnight at 37 °C with shaking at 150 rpm. Overnight cultures were serially diluted in double distilled autoclaved water and plated on LB agar to determine CFU/ml of recovered targeted bacteria. All experiments were performed in triplicates and repeated three times.

Statistical significance analysis was performed using an unpaired, two-tailed Student’s t-test (JMP software v.5; SAS Institute Inc., Cary, NC, U.S.A.). The CFU/ml of surviving bacteria, when co-cultured in the presence of alginate films, was compared to the no treatment control (alginate film with no chestnut extract). *P* values of less than 0.05 were considered to be statistically significant.

### Thermal analysis

Thermal and thermoxidative stability of neat sodium alginate and the sodium alginate films with chestnut extract and various plasticizers were investigated by thermogravimetric analysis (TGA) and differential scanning calorimetry (DSC).Thermogravimetric analysis was carried out using Mettler Toledo TGA 2 Thermo-balance. The sodium alginate film samples (about 5 mg) were heated up in an open platinum crucible (Pt 70 μL), in the temperature range from 30 to 800 °C at the heating rate β = 10 °C/min, in the dynamic (100 mL/min) nitrogen (inert atmosphere) or air (oxidative atmosphere) atmosphere. Two parameters were measured: the temperature of the onset of degradation (T_onset_), the temperature at maximum degradation (T_peak_) and weight loss on evaporation of water (∆m).The thermographs (thermogravimetric (TG) curves and derivative thermogravimetric (DTG) curves) were analyzed using the STARe Thermal Analysis Software.

Differential Scanning Calorimetry (DSC) measurements were performed using Mettler Toledo DSC 822e Differential Scanning Calorimeter. The alginate film samples (about 5 mg) were heated in an aluminum crucible (Al 40 μL) closed with perforated lid (0.5 mm), in dynamic (50 ml/min) nitrogen atmosphere, in the temperature range from 0 to 300 °C at the heating rate β = 10 °C/min.

### Chemical structure

Fourier transform infrared spectroscopy (FTIR) was used to identify the chemical structure of the sodium alginate films with chestnut extract and various plasticizers and possible interactions between their components. The FTIR spectra were measured using a Spectrum Two spectrometer (Perkin Elmer). The spectra were averaged for 25 scans recorded at a resolution of 2 cm^-1^ in the range from 4000 to 650 cm^−1^.

## Results and discussion

### Mechanical properties

**Figure 2:**
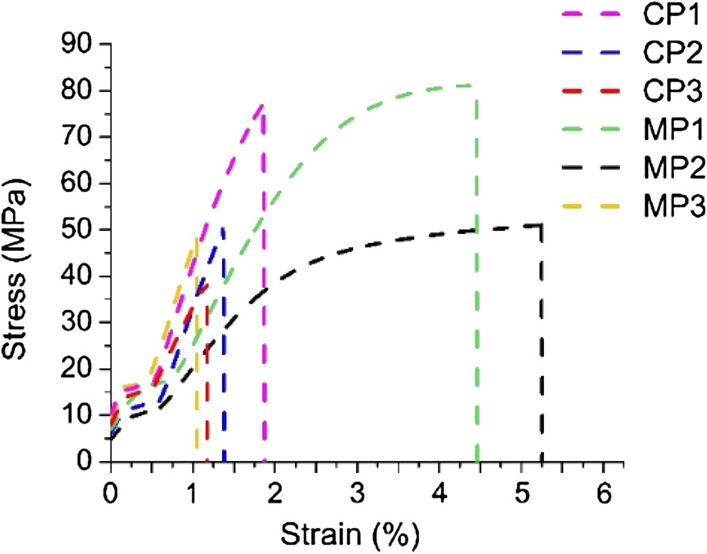
Effect of the bio-based plasticizers on tensile strength and elongation at break for sodium alginate films.

The effect of the bio-based plasticizers incorporated in the formulations of sodium alginate with chestnut extract on the tensile strength and elongation at break of the films was investigated. All films had a similar thickness of ca. 50 μm. Typical stress-strain curves are shown in Fig. 2. It can be seen that the highest tensile strength values, 75–76 MPa, exhibited films containing MP1 mixture and commercially available glycerol, sample CP1. The values are about 70% higher than the values for films with the other plasticizers. The lowest tensile strength value, ca. 35 MPa, was noted for CP3 sample, wherein epoxidized palm oil was used as a plasticizer. Almost equally low value was observed for MP3 sample with the epoxidized mixture of synthesized plasticizers. The highest values of elongation at break were recorded for the synthesized plasticizer mixtures, i.e. MP1 and MP2 (about 4.5% and 5.5%, respectively). In this case, the values were about 50% higher than for other samples investigated. Such significantly better elongation at break is related to the increased flexibility of the polymer chains and compatibility between polymer matrix and plasticizer. It was confirmed by SEM images (Fig. 5), wherein the structures of MP1 and MP2 samples are much more homogeneous than that of the other samples. Zactiti and Kieckbusch^62^ also studied sodium alginate-based films plasticized with glycerol and they obtained values of about 120 MPa and 4.5% for tensile strength and elongation at break, respectively. On the other hand, Paixão et al.^29^ studied sodium alginate films plasticized with glycerol, tributyl citrate or tributyl citrate/glycerol mixture. They noted that sodium alginate plasticized with tributyl citrate showed the highest tensile strength (about 150 MPa), while for the material plasticized with glycerol it was only ca. 104 MPa. In contrast, the elongation at break value was the highest for films plasticized with glycerol and it was equal to ca. 6%. Aadil and Jha^28^ proposed two plasticizers other than glycerol for sodium alginate, i.e. epichlorohydrin and polyethylene glycol. They found that the addition of the latter leads to an increase in the intermolecular space due to a decrease in the hydrogen bonds between lignin and sodium alginate, resulting in a decrease in glass transition temperature and melting point, which in turn improves the flexibility and processing properties of the film.

### Hydrophilic properties

**Figure 3:**
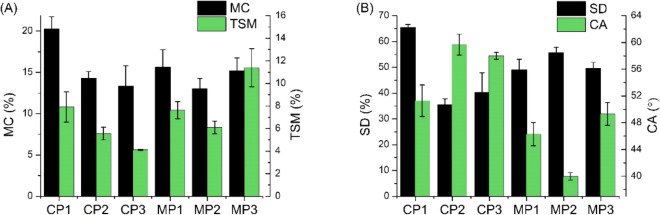
(**A**) Moisture content and total soluble matter; (**B**) swelling degree and water contact angle of the plasticized sodium alginate films.

Moisture content and total soluble matter values of the obtained films with chestnut extract and various plasticizers are shown in Fig. 3A. The results show that the highest MC value, about 20%, was recorded for the sample plasticized with glycerol, i.e. CP1. MC for the other samples was comparable, at around 15%. Analyzing the TSM values, the highest value for MP3, about 11%, and the lowest for CP3, about 3%, can be observed. Among the synthesized plasticizer mixtures, the lowest TSM value was recorded for MP2, at around 6%. In contrast, SD results, Fig. 3B, showed that CP1 has the highest swelling properties, about 65%, and the lowest was recorded for CP2 and CP3, about 40%. The results are similar to those obtained in others studies on sodium alginate films^63,64^. In contrast, Eltabakh et al.^65^ proposed sodium alginate maltodextrins-based films with phenolic extract of *Azolla pinnata* fern leaves for which slightly lower hydrophilicity results were obtained. In this case, a noteworthy effect was decrease in MC, SD and TSM values which was associated with the use of the phenolic extract. On the other hand, Abdin et al.^66^ proposed sodium alginate arabic gum based films with *Syzygium cumini* seeds extract. Also in this case, a decrease in the hydrophilic properties of the obtained films with the addition of the extract was noted. However, in both cases, the values were slightly lower for the control samples (without the extract) than the results obtained for the films investigated in our studies. This suggests that the lower hydrophilic character of the films was influenced primarily by the addition of maltodextrins and arabic gum. Contact angle studies confirmed that all of the obtained sodium alginate-based films are hydrophilic (contact angle < 90°). Nevertheless, significant differences were noted for samples containing different plasticizers. The most hydrophilic character was observed for MP2 (CA about 40°). It is worth noting that the epoxidation of the plasticizer caused a decrease in the hydrophilic character of the film, as its CA increased from 40° (MP2) to about 50° (MP3). Lu et al.^67^ prepared similar films based on sodium alginate crosslinked with CaCl_2_ and plasticized with glycerol but their recipes were enriched with nano-silica and oregano essential oil. The CA value of the resulting films was about 30°, which also indicates the hydrophilic nature of these films. A similar finding was observed by Hou et al.^68^, who used films prepared with sodium alginate and agar and modified with nano SiO_2_—the resulting CA value was about 60°. This suggests that neither CaCl_2_ crosslinking or presence of the forementioned plasticizers or nano additive change the nature of the films to hydrophobic.

### Barrier properties

**Figure 4:**
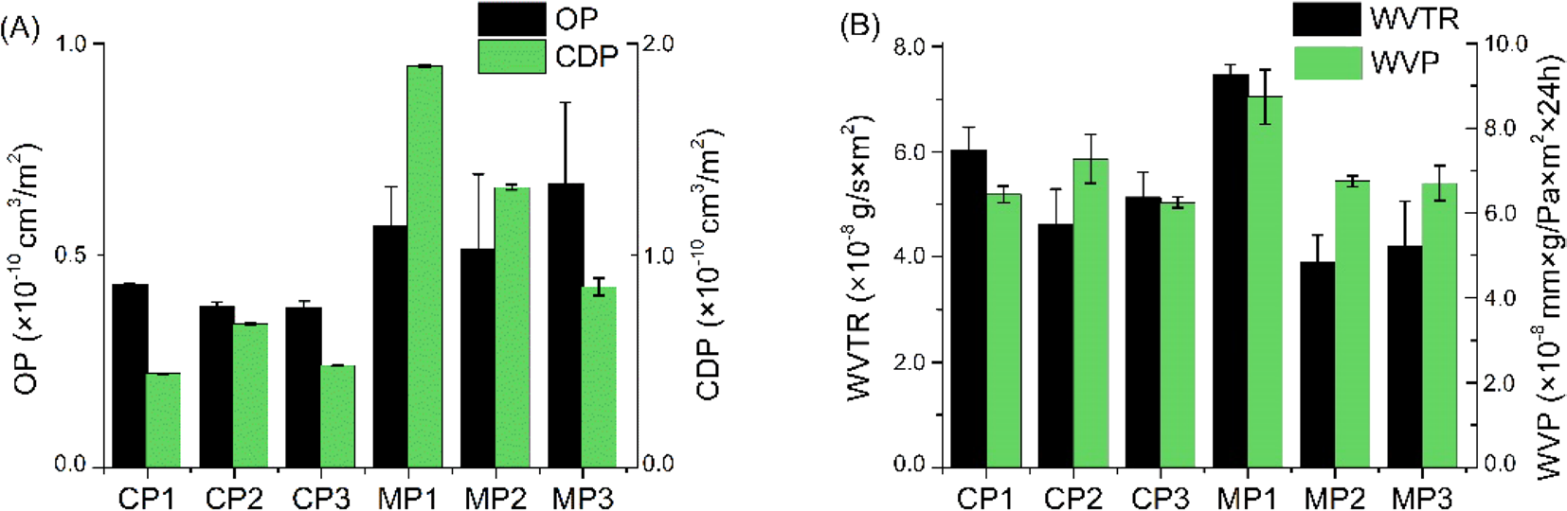
(**A**) Permeability of oxygen (OP) and carbon dioxide (CDP); (**B**) water vapor transmission rate (WVTR) and water vapor permeability (WVP) of the plasticized sodium alginate films.

The effect of different plasticizers on the barrier properties of the sodium alginate films with chestnut extract is shown in Figure 4. Many factors can affect the gas permeability of a film, such as its thickness, water sensitivity and crystallinity^69^. According to Jouki et al.^69^, polymers with high crystallinity, due to their ordered structure, show significantly lower gas permeability than polymers with low crystallinity. This is primarily because the amorphous phase of the polymer is mainly responsible for the mass transfer of gases. The oxygen permeability (OP) of food packaging materials is essential for food preservation^70,71^. As shown in Fig. 4A, the oxygen permeability values were relatively low for all samples (about 6 × 10^−11^ cm^3^/m^2^) compared to commercially available polylactide film^41^. Carbon dioxide barrier properties for films used for packaging are as important as OP because they affect respiration or oxidation reactions in food and also affect the shelf life of products^72^. In our case, CO_2_ permeability was also relatively low for all samples—about 5 × 10^−11^ cm^3^/m^2^. However, it is worth noting that epoxidation of the plasticizer led to a decrease in CDP. Despite the slightly higher OP and CDP for the films with the plasticizers synthesized in our study, their values were still lower or nearly equal than for the commercially available polylactide film, for which the values are on the order of 10^−9^ and 10^−10^, respectively^41^. Water vapor permeability (WVP) is another parameter that determines the water sensitivity of films. It plays an important role in the broad applications of biodegradable films^73^. In the present study, Fig. 4B, the overall WVP and WVTR increased slightly only for MP1, and for the other samples it was without significant changes compared to CP1.

### Surface morphology

**Figure 5:**
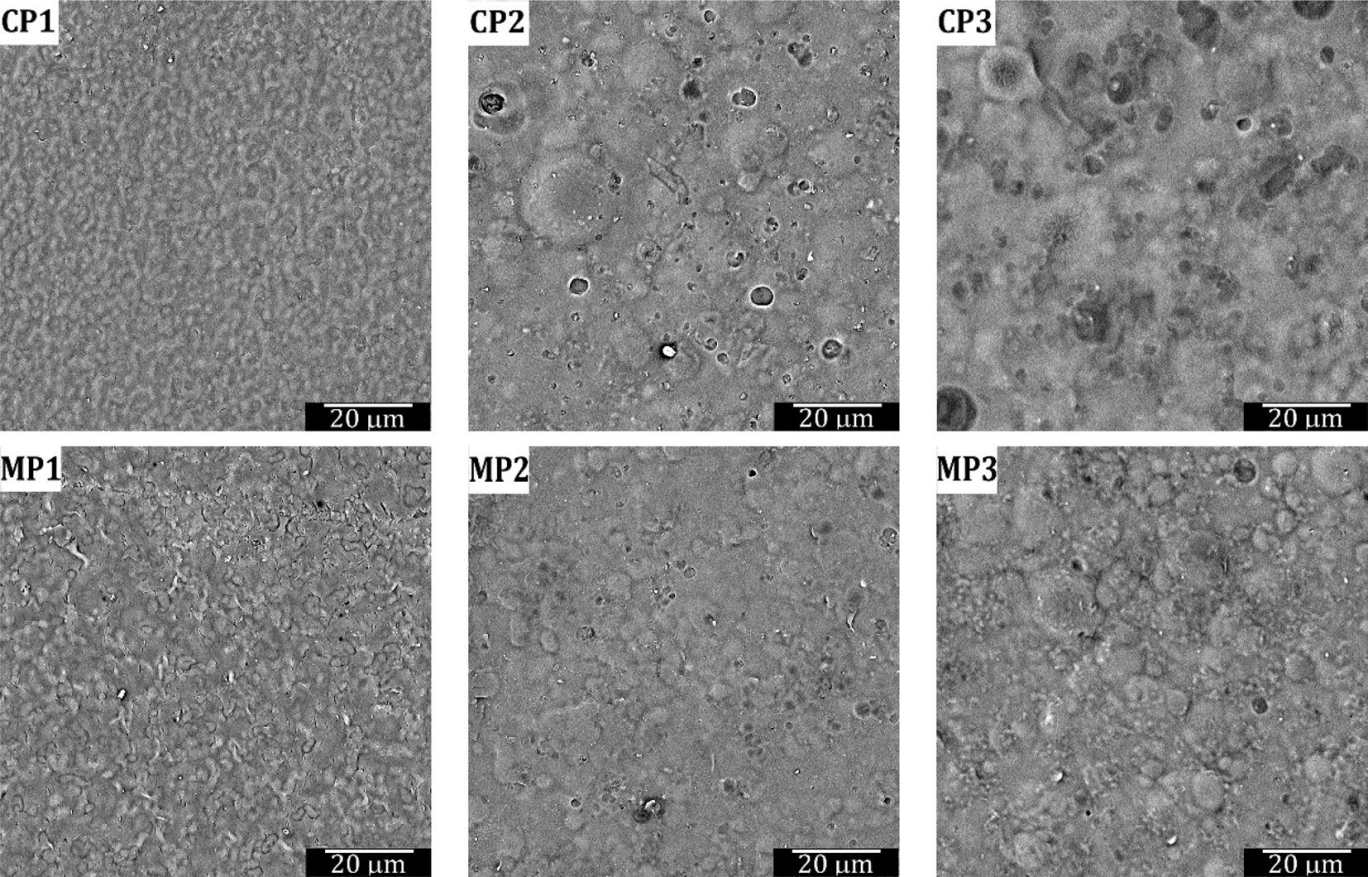
SEM images of sodium alginate film surfaces prepared with different plasticizers.

**Figure 6:**
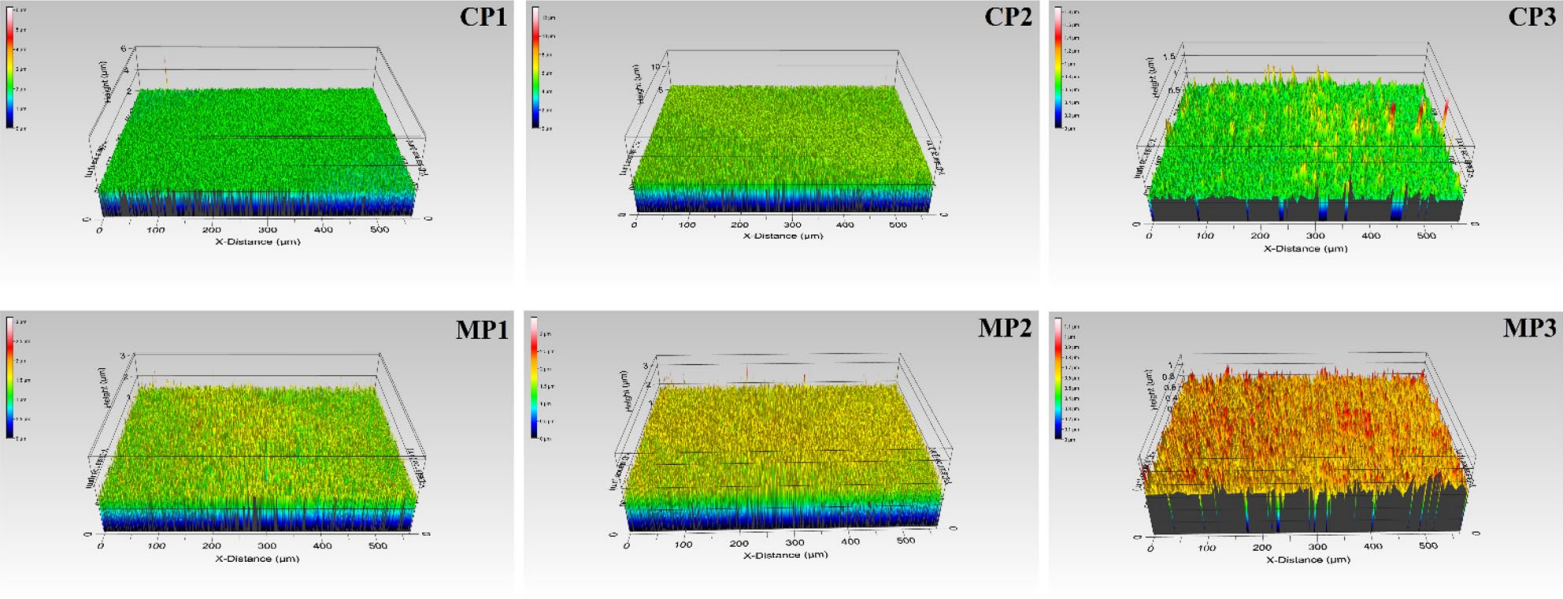
Optical profiles of sodium alginate film surfaces prepared with different plasticizers.

To evaluate the homogeneity and structure of the sodium alginate film surfaces, their SEM images and interferometric optical profiles are shown in Figs. 5, 6, respectively. It can be noted that all films are quite uniform in roughness, with the average S_a_ of 50 nm (Table 2). It can be noted that the films with glycerol (CP1) and with the two synthesized plasticizers (MP1 and MP2) presented a more homogeneous and uniform surface structure (as evidenced in their SEM images) than the films prepared with the other plasticizers under investigation, even though the smoothest surface is represented by MP3 (S_a_ = 38.7 ± 2.2 nm). This homogeneous structure mainly influenced the mechanical properties of the obtained sodium alginate-based films. They exhibited better tensile strength and elongation at break values compared to samples with a heterogeneous structure. Such a non-uniform surface with aggregate structures was observed for films prepared with epoxidized plasticizers (CP2, CP3 and MP3), what can be attributed to the limited compatibilityof the components with the polysaccharide matrix^72,74^. The observed difference in the morphology of the films was also in line with the gas barrier properties (the non-uniform surface and aggregates resulted in higher barrier properties).

**Table 2:** Surface roughness of sodium alginate film surfaces prepared with different plasticizers.

Symbol	CP1	CP2	CP3	MP1	MP2	MP3
Roughness (S_a_, nm)	51.8 ± 1.1	49.4 ± 0.7	55.3 ± 5.6	64.6 ± 4.5	56.5 ± 7.5	38.7 ± 2.2

### Antimicrobial activity

**Figure 7:**
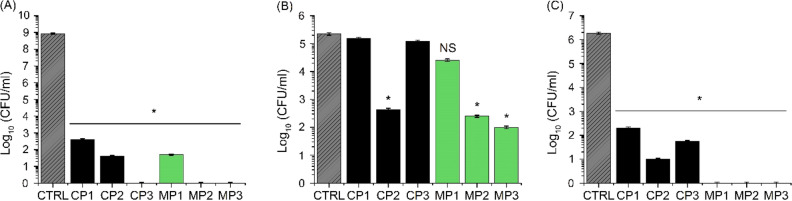
Antimicrobial activity of plasticized sodium alginate films against *E. coli* (**A**), *C. albicans* (**B**) and *S. epidermidis* (**C**). Asterisk represents statistically significant difference (*p* < 0.05). NS = no significance difference relative to the control (CTRL-plasticized sodium alginate film without chestnut extract).

The results of microbiological studies, Figs. 7A,C, demonstrate that all alginate-based films containing chestnut extract exhibited strong antibacterial activity against both gram-negative *E. coli* and gram-positive *S. epidermidis* with up to 7 to 8-fold reduction in the recovered CFU/ml, respectively. In the case of fungi *C. albicans*, only a two–three-fold inhibition relative to the control disc was observed, Fig. 7B. Interestingly, the highest antibacterial effects were shown by sodium alginate films containing chestnut extract and synthesized plasticizers, particularly MP2 and MP3, which were found to decrease the bacterial presence to single cells (Table S1, Supplementary). The results are in line with previous research^75–77^ showing antimicrobial properties of fatty acid esters. In the case of alginate films containing both chestnut extract and fatty acid based plasticizers, a synergistic effects are observed in relation to their antimicrobial activity. Even though chestnut extract-containing sodium alginate films were not found to exhibit as strong antifungal properties as antibacterial ones, still, a significant decrease in the number of cells was observed on the surface of alginate films plasticized with CP2, MP2 and MP3. These observations can be also attributed to the presence of fatty acids in the plasticizers used for the fabrication of materials. Although chestnut extract does not exhibit strong antifungal properties, fatty acids and their esters are known to be efficient antifungal agents^77^. Since soybean oil contains higher content of linoleic acid (characterized by a low minimum inhibitory concentration^78^ than palm oil, also CP2 is more active against *C. albicans* than CP3.

### Thermal analysis

**Figure 8:**
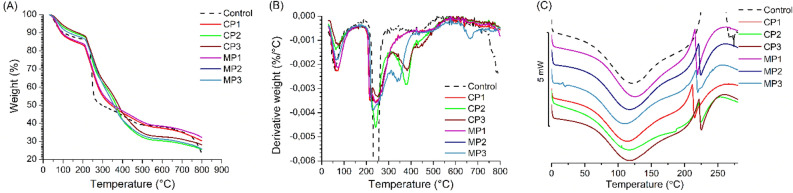
Thermal curves of neat sodium alginate and plasticized sodium alginate-based films: (**A**) TG in nitrogen; (**B**) DTG in nitrogen; (**C**) DSC, where Control- neat sodium alginate.

The effect of various plasticizers on the thermal properties of sodium alginate films with chestnut extract was evaluated by thermogravimetric analysis and differential scanning calorimetry. TG and DTG curves in nitrogen atmosphere are shown in Fig. 8A,B, respectively. It can be seen that for all samples the first peaks in the DTG curves, below 200 °C, correspond to the evaporation of physically absorbed water. For neat sodium alginate (Control), the second weight loss is around 230 °C (T_onset_ in TG curves), while the maximum of degradation occured at 235 °C (T_peak_ in DTG curve), which corresponds to the decomposition of polysaccharides by the fracture of glycosidic bonds, decarboxylation, decarbonylation and loss of bonded water. The final degradation occured at around 500 °C, which might be attributed to the degradation of the formed intermediate compounds in the second stage and char formation^19,79^. For the plasticized alginate films, the degradation process has slightly decreased. Initial decomposition temperature (T_onset_) has decreased, compared to pure sodium alginate. Moreover neat alginate degradation gave a sharp peak while for plasticized films degradation peak was broader and a shoulder appeared indicating that the crosslink structure is shifting the alginate degradation to relatively higher temperatures. As shown in Table S2 (Supplementary Data), a similar relationship was observed in air atmosphere. Hence, these findings indicate that the addition of plasticizers retarded the initials thermal degradation and prolongs the degradations process^26^. Furthermore, the CP1 and MP1 films showed the lowest T_onset_ values, respectively 207.4 °C and 208.9 °C. This suggests in CP1 and MP1 samples the plasticizers could easily locate into alginate network and disrupt the intermolecular interactions. These disorders cause softening of the polymer structure, which results in increased chain mobility, making less packed chains more susceptible to degradation processes^8,26^.

Figure 8C shows DSC results. Thermograms for all samples showed a broad endothermic peak centered at 120 °C, confirming the evaporation of adsorbed water. The intensity of this DSC peak changes, what indicates a different amount of adsorbed water, but does not affect the hydrogen bonds between alginate molecules. The thermograms illustrate also the emergence of a sharp endothermic peak at 220 °C, probably corresponding to cleavage of calcium–carboxylate bonds within the complex. Such sharp endothermic peak indicates a highly ordered molecular arrangement forming the so-called ‘‘egg-box’’ structure within calcium alginate matrix^80,81^. Moreover, calcium crosslinking led to upward shift of the exothermic band from 240 to 281 °C indicative for increased alginate resistance to thermal oxidation^20^. However, no significant relationship between the type of plasticizer and the DSC curves shape was observed.

### Chemical structure

**Figure 9:**
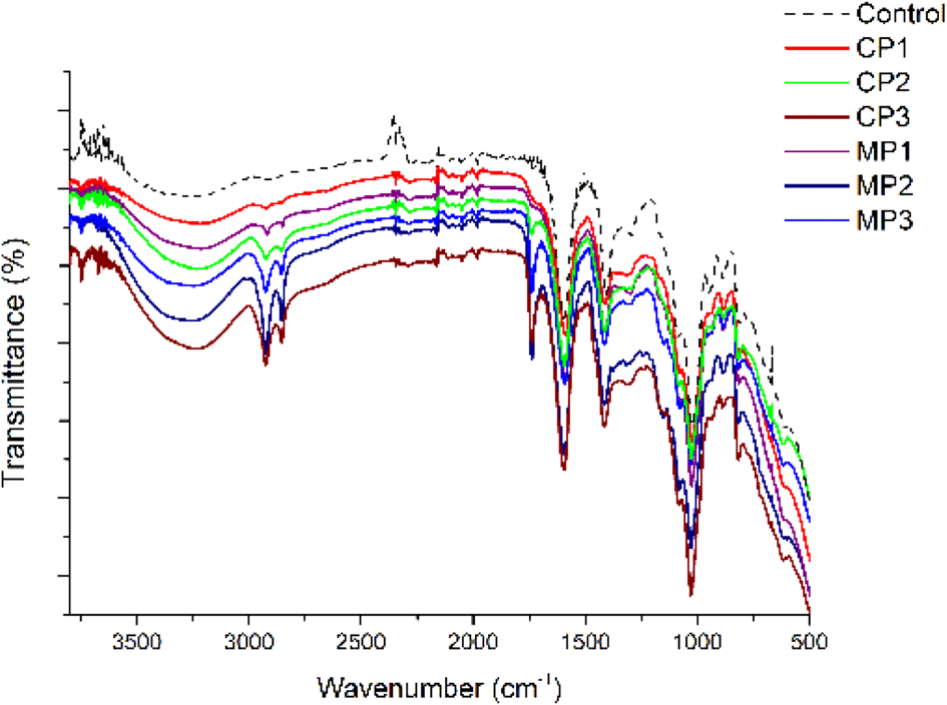
FT − IR spectra of sodium alginate films with different plasticizers, where Control- neat sodium alginate.

The FT-IR spectra of unplasticized and plasticized sodium alginate films with chestnut extract are shown in Fig. 9. The characteristic peaks of neat sodium alginate (Control) were observed at 3606–3018 cm^−1^ (broad, attributed to O–H stretching vibrations), 2975–2913 cm^−1^ (–CH stretching vibrations), 1604 cm^–1^ (asymmetric –COO– vibrations) and 1415 cm^−1^ (symmetric -COO- vibrations) and 1090–1017 cm^–1^ (C–O–C antisymmetric stretching vibrations)^24,82^. The absorbance bands at 1200–960 cm^−1^ attributed to the skeletal vibrations of pyranose ring of alginate^83^. Plasticized alginate films showed the same FT-IR patterns as the unplasticized ones. For the plasticized alginate films, a characteristic broad band was observed between 3700 and 3000 cm^−1^, which was attributed to the O–H stretching vibration. The intensity of this band increased with the addition of a plasticizer; the highest intensities were observed for CP3 and MP2, while the lowest for CP1 and MP1, suggesting hydrogen bonding between the plasticizer and alginate matrix. The bands at ca. 1600 cm^−1^ and 1405 cm^−1^, which were observed for each plasticized film, are characteristic for asymmetric and symmetric stretching vibrations of the COO– groups, respectively^84^. For CP2, CP3, MP2 and MP3, a band at 1748–1741 cm^−1^ was observed which can be attributed to the double carbonyl bonds (C=O) formation; the most intense peak was observed for MP2. The least intesive bands at 1758–1754 cm^−1^ were observed for CP1 and MP1, which also correspond to C=O stretching vibrations^28^. For CP1, C–H stretching vibration bands were observed at 2975 cm^−1^, while for the other samples at 2920 cm^−1^ and 2850 cm^−1^. The most intensive bands were observed for CP3, MP2 and MP3, confirming the presence of hydrocarbon chains in the structure of the used plasticizers (they contain fatty acids)^59^. The bands at 1090 cm^−1^ and 1020 cm^−1^ were attributed to C–O-C antisymmetric stretching vibrations present in all plasticized alginate films.

## Conclusion

In order to develop new antibacterial and environmentally friendly sodium alginate-based films for food packaging, the selection of proper plasticizer is essential. The obtained results confirmed that each investigated plasticizer modifies the structure of the polymer but in a different way. Films CP1, MP1 and MP2 showed a more homogeneous structure, suggesting that the plasticizers are more compatible with sodium alginate than the epoxidized ones (CP2, CP3 or MP3). It was found that the film plasticized with mixed esters of propylene glycol and acetic acid of propylene glycol (MP1) and the film plasticized with glycerol (CP1), showed better mechanical properties (tensile strength and elongation at break) than the other films studied in this paper. It was due to a more homogeneous structure, that did not show morphological changes with the appearance of agglomerations, which promote increased film brittleness. All the films showed a hydrophilic character (contact angle < 90°) and high barrier properties to oxygen, carbon dioxide and water vapor compared to commercially used polylactide or polyethylene films. In addition, the presence of plasticizers, both commercially available or synthesized ones, was found to increase the antibacterial activity of sodium alginate films with chestnut extract for both gram-negative and gram-positive bacterial strains. The highest antibacterial activity showed sodium alginate films containing chestnut extract and the synthesized plasticizer mixtures, particularly MP2 and MP3, which was due to the synergistic effects between chestnut extract and fatty acid-derived plasticizers in relation to their antimicrobial activity. As the sodium alginate-based films plasticized with the synthesized MP1 and MP2 showed better elongation at break and significantly better antibacterial properties compared to glycerol, further work is planned with these plasticizers to improve the mechanical properties of the films even more. Expanded research is also planned in terms of their application in the food packaging sector, such as migration studies of the plasticizers and the active compound, among others. In addition, the obtained results suggest the need to look for other additives that could enhance low hydrophobicity of the sodium alginate-based films.

## Supplementary Information

Supplementary Information.

## Data Availability

The datasets used and/or analysed during the current study available from the corresponding author on reasonable request.
