# Supplementary material for: Modulation of physicochemical properties and antimicrobial activity of sodium alginate films through the use of chestnut extract and plasticizers
Source: Sci Rep. 2023 Jul 17;13:11530. doi: 10.1038/s41598-023-38794-3 (PMC10352291; doi:10.1038/s41598-023-38794-3)
Supplement: Supplementary file 1 — Supplementary Information. [file 41598_2023_38794_MOESM1_ESM.docx]

# SUPPLEMENTARY DATA

**Modulation of Physicochemical Properties and Antimicrobial Activity of Sodium Alginate Films through the Use of Chestnut Extract and Plasticizers**

Weronika Janik, Michał Nowotarski, Kerstin Ledniowska, Divine Yufetar Shyntum, Katarzyna Krukiewicz, Roman Turczyn, Ewa Sabura, Simona Furgoł, Stanisław Kudła, and Gabriela Dudek





**Figure S1.** Schematic route of the synthesis of mixed esters of propylene glycol and acetic acid (sample MP1)





**Figure S2.** Schematic route of the synthesis of mixed esters of propylene glycol, oleic acid and succinic acid (sample MP2)





**Figure S3.** Schematic route of the synthesis of epoxidized mixed esters of propylene glycol, oleic acid and succinic acid (sample MP3)

**Table S1.** Number of colony-forming units on the surface of alginate films and control (empty well plate)

|  | ***Escherichia coli*** | ***Candida albicans*** | ***Staphylococcus aureus*** |
| --- | --- | --- | --- |
| **Control** | 850000000 | 222000 | 1850000 |
| **CP1** | 410 | 153400 | 200 |
| **CP2** | 42 | 436 | 10 |
| **CP3** | 1 | 122000 | 55 |
| **MP1** | 51 | 25644 | 1 |
| **MP2** | 1 | 249 | 1 |
| **MP3** | 1 | 100 | 1 |

**Table S2.** Results of the thermal properties, where Control-neat sodium alginate

|  | ***Δm /% (m/m)*** | ***T_onset_ /°C*** | ***T_peak_ /°C*** |
| --- | --- | --- | --- |
| **Nitrogen** | | | |
| **Control** | 13,1 | 229,0 | 246,0 |
| **CP1** | 15,7 | 207,4 | 239,7 |
| **CP2** | 10,9 | 218,3 | 241,3 |
| **CP3** | 10,2 | 214,3 | 250,2 |
| **MP1** | 15,0 | 208,9 | 242,2 |
| **MP2** | 12,7 | 215,3 | 233,2 |
| **MP3** | 12,0 | 215,5 | 251,5 |
| **Air** | | | |
| **Control** | 13,4 | 230,2 | 247,2 |
| **CP1** | 16,6 | 207,1 | 240,2 |
| **CP2** | 11,9 | 215,2 | 240,2 |
| **CP3** | 12,3 | 211,6 | 245,2 |
| **MP1** | 15,0 | 208,7 | 247,7 |
| **MP2** | 13,5 | 215,0 | 228,3 |
| **MP3** | 11,3 | 214,0 | 254,5 |
